# First person – Lata Adnani

**DOI:** 10.1242/bio.039610

**Published:** 2018-11-15

## Abstract

First Person is a series of interviews with the first authors of a selection of papers published in Biology Open, helping early-career researchers promote themselves alongside their papers. Lata Adnani is first author on ‘Plag1 and Plagl2 have overlapping and distinct functions in telencephalic development’, published in BiO. Lata conducted the research described in this article while a PhD student in Dr Carol Schuurmans’ lab at University of Calgary, Canada. She is now a postdoctoral fellow in the lab of Dr Janusz Rak at McGill University Health Centre, Montreal, Canada, investigating neuroscience.


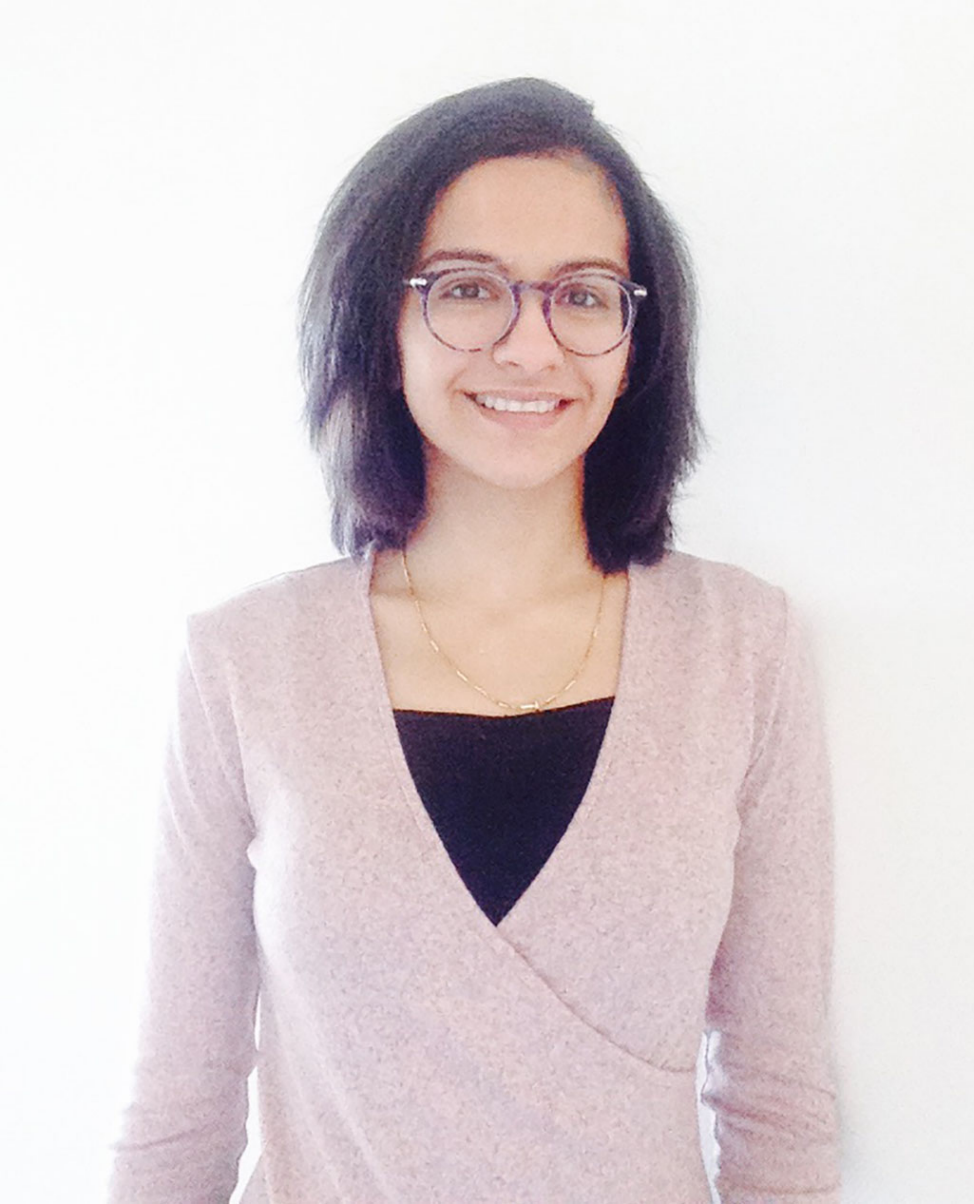


**Lata Adnani**

**What is your scientific background and the general focus of your lab?**

Before joining the Schuurmans’ laboratory, I completed a BSc Honors in the Biotechnology degree program at Manipal University, Dubai Campus in the United Arab Emirates. During my undergraduate degree, I worked on understanding the influence of hypertension on diabetes and vice versa. Working on this project exposed me to molecular biology and an interest in neuroscience encouraged me to pursue a PhD in the laboratory of Dr Carol Schuurmans at the University of Calgary (later moved to the Sunnybrook Research Institute). The Schuurmans’ laboratory focuses on understanding cell fate specification in the neocortex and retina during development and in disease. The development of a functional central nervous system (CNS) requires that an appropriate number of correct cell types are first patterned precisely, proliferate followed by temporally differentiate, and then migrate to their proper destinations for establishing specific synaptic connections. Long-term cognitive and behavioral deficits can arise when patterning, neurogenesis, neuronal migration or circuit formations are disrupted during development. The focus of my doctoral research in Schuurmans’ lab was on understanding neural cell fate decisions, cell migration and the specification of cell fates in the developing neocortex. Specifically, I focused on members of the Plag family of transcription factors, including *Plag1, Plagl1* (also known as *Zac1*) and *Plagl2*. I first demonstrated that *Zac1* regulates neuronal migration and neuronal morphology during neocortical development (Adnani et al., 2015). I then studied *Plag1* and *Plagl2*, which are also expressed in the developing neocortex*,* demonstrating non-overlapping roles for these genes in establishing regional boundaries and proliferation in the developing telencephalon (the subject of this study).

**How would you explain the main findings of your paper to non-scientific family and friends?**

The neocortex is the region of the brain involved in regulating higher order cognitive functions such as learning and memory. Understanding how this region of the brain develops is important for deciphering how the brain is malformed in neurodevelopmental disorders, and for devising strategies to replace neurons when they are lost in neurodegenerative disease. My PhD work focused on a gene family known as the Pleomorphic adenoma (Plag), which includes three genes: *Plagl1* (also known as *Zac1*), *Plag1* and *Plagl2*. All of these genes had previously been studied for their role in tumor formation – *Zac1* inhibits tumor formation whereas *Plag1* and *Plagl2* promote tumor formation. Schuurmans’ lab previously demonstrated that all three of these genes are also expressed in dividing progenitor cells in the developing neocortex, and my project was to characterize their functions in this region of the developing brain. In this new publication in Biology Open, I demonstrated that *Plag1* and *Plagl2* play redundant roles in embryonic survival; deletion of both *Plag1* and *Plagl2* is not compatible with life, resulting in early embryonic lethality suggesting compensatory roles for both these genes during development. In addition, I demonstrated that altering *Plagl2*, but not *Plag1*, induced a patterning defect in the developing brain, while *Plagl2* influenced the ability of embryonic cells to divide. In summary, my studies identify two new players that are important for guiding the proper formation of the embryonic neocortex, and these genes may be important for understanding how neurodevelopmental defects might arise in diseases such as autism spectrum disorder.

**What are the potential implications of these results for your field of research?**

We have identified novel roles for *Plag1* and *Plagl2* in neocortical development which have otherwise been implicated in tumorigenesis. These *Plag* genes have some overlapping functions in embryo survival as we were unable to obtain double *Plag1;Plagl2* knockout embryos during early development. We have determined that *Plagl2* is a new player in regulating the formation of dorsal-ventral borders in the telencephalon, and it may interact with other known players in this pathway (e.g. Pax6, Gsx2, etc). We also observed a decrease in cell proliferation and an increase in neuronal differentiation in the *Plag1* mutant embryos at E12.5, suggesting that *Plag1* maintains the balance between cell proliferation and differentiation early in neocortical development. Taken together, our analyses of *Plag* gene function may be important for understanding cognitive malfunctions such as learning disabilities and autism spectrum disorder. To sum up, our findings are building blocks towards understanding the molecular basis of how a functional neocortex develops.

“Our analyses of *Plag* gene function may be important for understanding cognitive malfunctions such as learning disabilities and autism spectrum disorder.”

**What, in your opinion, are some of the greatest achievements in your field and how has this influenced your research?**

There are many brilliant accomplishments in the field of neuroscience that have inspired me to be more creative, yet pragmatic, in research. I remember being extremely fascinated reading about the *Reeler* mice and how one gene could dramatically alter the formation of the neocortical layers. This was one of the many articles that made me passionate about neuroscience. Studies such as: the generation of an optogenetic tool to track single cells in action so as to determine how a cell functions upon being stimulated; development of a technique called CLARITY, to generate transparent brains which bestows upon us the ability to observe a multitude of cell connections throughout the brain; regeneration of vision using skin-derived stem cells in humans; development of organoids to mimic different organ complexities, which can potentially revolutionize drug discovery; detection of the importance of extracellular vesicles in cancer, which can be used as potential biomarkers and drug carriers; the capability of profiling single cells, which gives us the ability to hear what the heterogenous pool of cancer cells talk; and similar research have influenced me to ask big picture questions, thereby molding my way of thinking considerably. Such innovative studies have enabled me to take small steps towards significantly contributing to solving a question that has been haunting us for generations: ‘how do we cure cancer?’ This is the subject of my current postdoctoral work.

“Innovative studies have enabled me to take small steps towards significantly contributing to solving a question that has been haunting us for generations: ‘how do we cure cancer?’”

**What's next for you?**

Applying my research knowledge to a disease model (i.e., glioma) at the end of my PhD career was very rewarding, and brain cancer is a research area that I am currently pursuing for my postdoctoral training. I have recently started working in the laboratory of Dr Janusz Rak at McGill University as a postdoctoral fellow. The Rak laboratory is very interested in understanding not only angiogenesis but also the importance of extracellular vesicles in glioblastoma and in other gliomas affecting patients. Switching fields from neurodevelopment to neurooncology has been quite exciting and I am learning new things everyday. I aspire to be an accademic researcher and to have a laboratory of my own where I can bring together the knowledge I am obtaining, and to grow my expertise, to be able to connect the vast fields of biochemistry, molecular biology, neuroscience and neurooncology.
